# Emotion Recognition of Chinese Paintings at the Thirteenth National Exhibition of Fines Arts in China Based on Advanced Affective Computing

**DOI:** 10.3389/fpsyg.2021.741665

**Published:** 2021-10-22

**Authors:** Jing Li, Dongliang Chen, Ning Yu, Ziping Zhao, Zhihan Lv

**Affiliations:** ^1^College of Art, Qingdao Agricultural University, Qingdao, China; ^2^College of Computer Science and Technology, Qingdao University, Qingdao, China; ^3^Faculty of Arts, Uppsala University, Uppsala, Sweden

**Keywords:** advanced affective computing, Chinese paintings, deep learning, the Thirteenth National Exhibition of Fines Arts in China, emotion recognition

## Abstract

Today, with the rapid development of economic level, people’s esthetic requirements are also rising, they have a deeper emotional understanding of art, and the voice of their traditional art and culture is becoming higher. The study expects to explore the performance of advanced affective computing in the recognition and analysis of emotional features of Chinese paintings at the 13th National Exhibition of Fines Arts. Aiming at the problem of “semantic gap” in the emotion recognition task of images such as traditional Chinese painting, the study selects the AlexNet algorithm based on convolutional neural network (CNN), and further improves the AlexNet algorithm. Meanwhile, the study adds chi square test to solve the problems of data redundancy and noise in various modes such as Chinese painting. Moreover, the study designs a multimodal emotion recognition model of Chinese painting based on improved AlexNet neural network and chi square test. Finally, the performance of the model is verified by simulation with Chinese painting in the 13th National Exhibition of Fines Arts as the data source. The proposed algorithm is compared with Long Short-Term Memory (LSTM), CNN, Recurrent Neural Network (RNN), AlexNet, and Deep Neural Network (DNN) algorithms from the training set and test set, respectively, The emotion recognition accuracy of the proposed algorithm reaches 92.23 and 97.11% in the training set and test set, respectively, the training time is stable at about 54.97 s, and the test time is stable at about 23.74 s. In addition, the analysis of the acceleration efficiency of each algorithm shows that the improved AlexNet algorithm is suitable for processing a large amount of brain image data, and the acceleration ratio is also higher than other algorithms. And the efficiency in the test set scenario is slightly better than that in the training set scenario. On the premise of ensuring the error, the multimodal emotion recognition model of Chinese painting can achieve high accuracy and obvious acceleration effect. More importantly, the emotion recognition and analysis effect of traditional Chinese painting is the best, which can provide an experimental basis for the digital understanding and management of emotion of quintessence.

## Introduction

Now that the world comes to the big data era, Artificial Intelligence (AI) has impacted people’s lives profoundly; the traditional interpersonal interactions begin to shift to human-computer emotional interactions. In the meantime, people put forward higher requirements for esthetic appreciations than in the past. The performance of simple painting techniques has been unable to meet the emotional needs of artists, more and more artists focus on the emotional expression of works. In this case, how to understand the emotional expression of works of art is particularly important. More and more researchers are committed to the emotional expression of works of art.

The works of the Thirteenth National Exhibition of Fines Arts are close to the daily life of the Chinese people. It reflects the new changes in the development of modern society and the achievements of China’s development in the new era, and shapes and depicts the spirit of the Chinese nation in the new era. The Chinese paintings in this exhibition have the following emotional descriptions: Li Encheng’s *Fanghua* shows the posture of natural mountains and fields, praising the unknown and selfless workers in all walks of life. *Mission* embodies the patriotic spirit of heroism and model practitioners—fire fighters. *Pursuing the Dream of Space* shows the astronauts’ exploration of the secrets of the universe, the endless Chinese spirit, and Chinese power. The selected works of Chinese painting in the Thirteenth National Exhibition of Fines Arts reveal that, the themes consciously highlight the spirit of the times, depict all kinds of people’s livelihood, the Chinese mountains and rivers are thriving, and the painting themes show a new fashion of the times.


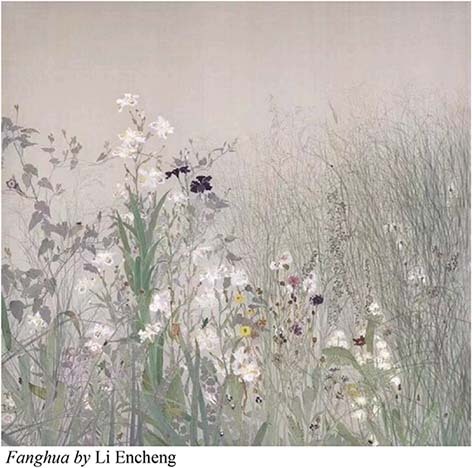



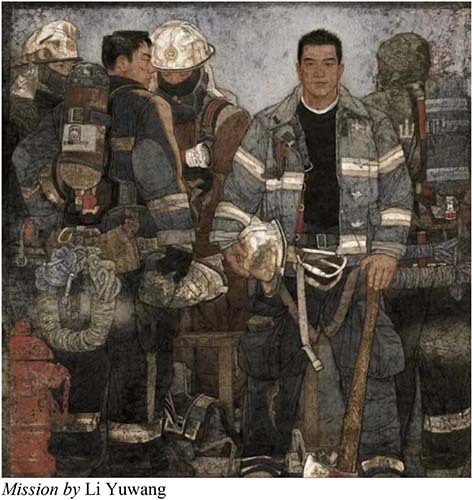



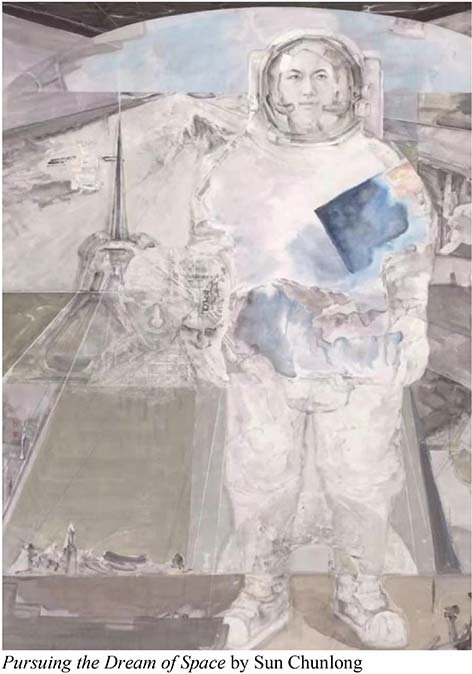


It may be challenging to capture the emotions expressed in these paintings accurately. Therefore, using Advanced Affective Computing to analyze the emotions expressed in these paintings can offer an opportunity for the general public to appreciate art. However, the result may be contrary to the user’s actual emotion applying the traditional single model for Chinese painting sentiment analysis. Regarding the complementary associations of various modality information, multimodal emotion recognition has gradually received widespread attention to thoroughly utilize the present modality data to capture information in and between the modalities (He and Zhang, 2018). Users pose new challenges to emotion recognition while they employ different modalities to portray their real-time emotions and make their current emotions vivid.

As for image data such as Chinese paintings, features such as SIFT (Scale-Invariant Feature Transform) operator vectors can be obtained. Original feature data of different modalities have their unique structural characteristics belonging to different feature spaces. Hence, noise modality may also be mixed into them during feature extraction (Zhang et al., 2020). Traditional emotion recognition algorithms may increase the calculation time and space cost, resulting in disturbed recognition outcomes. As of now, many large-scale, high-quality datasets are proposed, such as ImageNet, Deep Learning (DL) approaches have made breakthrough achievements in image recognition. Compared with artificially designed features, the unique multi-layer structure of DL enables it to learn the in-depth features that gradually transits from universal, low-level visual features (such as edges and textures) to high-level semantic representations (such as the torso and head); the higher the level, the stronger the expression (Wang et al., 2019; Chen et al., 2020). The hierarchical nature of the deep features provides a practical means to bridge the semantic gap and understand human affects in paintings (Liu et al., 2021).

To sum up, under the trend of the rapid development of AI, using advanced affective technology to identify the emotion of Chinese painting with multiple feature spaces has become the internal driving force for people to understand artistic emotion, which has great practical significance for the inheritance of Chinese traditional culture. Therefore, the innovation is to solve the problem of “semantic gap” in the emotion recognition task of images such as traditional Chinese painting, select and improve the AlexNet algorithm on the basis of deep learning theory to further improve its performance. The study also adds chi square test to solve the data redundancy and noise problems in various modes such as Chinese painting. Additionally, a multimodal emotion recognition model of Chinese painting based on improved AlexNet neural network and chi square test is designed to provide theoretical support for the digital understanding and development of emotion in Chinese quintessence.

## Related Works

### Trend of Intelligent Emotion Recognition

Emotion capture is the foundation of daily communication. Multi-party cooperation in all professions and trades is inseparable from emotion analysis and emotion recognition. Jain et al. (2018) proposed a hybrid DNN (Deep Neural Network) to recognize emotions in face images. They tested this hybrid DNN on two public datasets and harvested satisfactory experimental results (Jain et al., 2018). Avots et al. (2019) captured and classified facial expressions using SVMs (Support Vector Machines). They utilized Viola-Jones facial recognition algorithm and CNN (AlexNet) emotion classification algorithm to find human faces in keyframes. Ultimately, the proposed algorithm was validated (Avots et al., 2019). Hwang et al. (2020) put forward a novel emotion recognition approach based on CNN (Convolutional Neural Network) while preventing local information loss. Lastly, visualization proved that this approach outperformed other Electroencephalography (EEG) feature representation models based on standard features, proving its effectiveness (Hwang et al., 2020). Wei et al. (2021) designed an algorithm to extract keyframes from videos based on emotion saliency estimation regarding the current status of video emotion recognition. Keyframes could be extracted to avoid the influence of the emotion-independent frames on the result by estimating the emotional saliency of the video frame. Through simulation, the designed algorithm could improve the performance of video emotion recognition, outperforming the present user-generated video emotion recognition (Wei et al., 2021).

### Advanced Affective Computing for Multimodal Analysis

Hammad et al. (2018) put forward a secure multimodal biometric system based on CNN and a QG-MSVM (Q-Gaussian Multi-Support Vector Machine) based on different fusion levels. They applied this internal fusion system to combine the biological characteristics to generate a biological characteristic template. Results demonstrated that the proposed system could provide higher efficiency, robustness, and reliability than the present multimodal authentication system (Hammad et al., 2018). Ćosić et al. (2019) enhanced the process of ATC (Air Traffic Controller) selection based on the traditional ATC psychophysiological data measurement, including complicated physiological, eye volume, and voice measurements and appropriate metrics, as well as the ability to measure the multimodality in particular stimulus tasks. They comprehensively analyzed the multimodal features of this method under different experimental conditions and found it pretty advantageous (Ćosić et al., 2019). Yuan et al. (2020) designed and implemented the MSNVRFL (Multimodal Sensing Navigation Virtual and Real Fusion Laboratory) regarding the importance of virtual experiments in HCI (Human-Computer Interaction). MSNVRFL was equipped with a set of experimental devices with cognitive functions, in which a multimodal chemical experiment fusion algorithm was explored, validated, and applied (Yuan et al., 2020). Walia and Rohilla (2021) reviewed the various biological activity detection technologies based on multimodal biometric systems. After analysis and discussion, they proposed a new classification approach and finally proved its effectiveness (Walia and Rohilla, 2021).

Apparently, most works about image sentiment analysis are based on face images or sequences; in contrast, the sentiment analysis for images such as Chinese paintings is still a great challenge. Works about intelligent Chinese painting recognition have been reported; nevertheless, they focused on identifying the author of the painting and analyzing the work style, with very little research on the emotions shown by Chinese paintings. Intelligent algorithms, such as DL, have not played a significant role in emotion recognition. In this case, DL approaches are employed in the present work to identify and analyze the emotions expressed in Chinese paintings, which is of great significance for the in-depth understanding of the emotions of Chinese paintings subsequently.

## Multimodal Emotion Recognition and Analysis of Chinese Paintings at the Thirteenth National Exhibition of Fines Arts in China Based on DL Approach

The Thirteenth National Exhibition of Fines Arts vividly shows the spirit of the Chinese nation in the new era and the happy life of the people. A DL-based multimodal emotion recognition model for Chinese paintings is proposed based on image data of Chinese paintings at the Thirteenth National Exhibition of Fines Arts in China. Subsequently, its performance is validated through comparative simulation experiments.

### Demand for Chinese Painting Emotion Recognition

**FIGURE 1:**
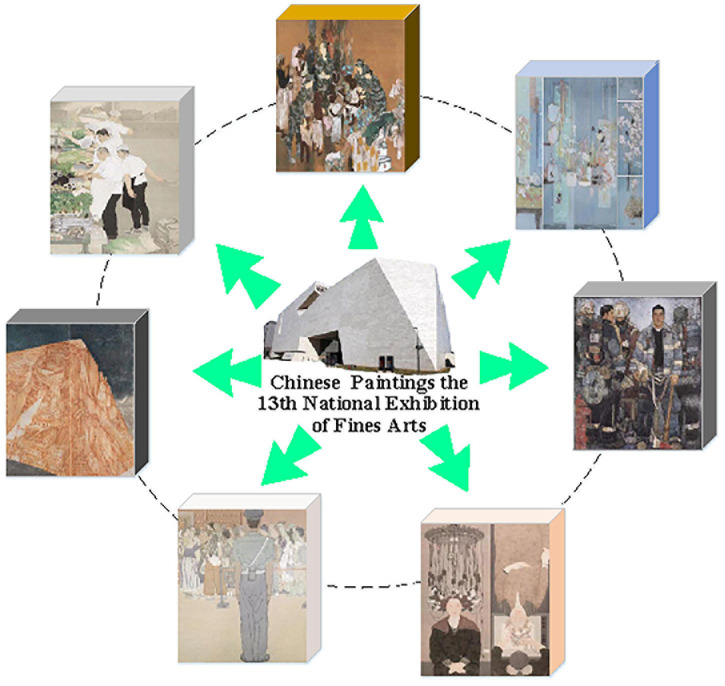
A demonstration of recognizing human affects in Chinese paintings at the Thirteenth National Exhibition of Fines Arts in China.

Chinese paintings pay attention to image modeling. It cannot blindly imitate the objective reality, nor can it be purely fabricated, but pursues the artistic state of “like and not like.” Based on respecting the objective images, painters integrate their feelings into the objective things. In this way, they can not only express their feelings in the works, but also make the images have emotion and vitality. Emotion is the driving force of Chinese painting creation (Bi et al., 2019; Yang et al., 2021). Traditional algorithms to recognize human affects in Chinese paintings often combine art theory and computer vision, practicing artificially designed features and statistical ML (Machine Learning) approaches to recognize the emotional responses evoked by Chinese paintings. The emotional response of Chinese paintings is presented in Figure 1. However, the large-scale, high-quality labeled datasets, such as ImageNet, make traditional ML approaches incapable of training deeper DL models, leading to severe overfitting.

Regularly, the large-scale dataset distribution used for pre-training is very different from the dataset distribution of the target task. For instance, the ImageNet dataset is dominated by natural images (such as animals and natural scenes). In contrast, image sentiment classification datasets such as Chinese paintings are completely composed of painting elements. Thus, if the pre-training weights of the ImageNet dataset are directly transferred, under-fitting caused by cross-domain transfer will occur. Meanwhile, a large-scale, image emotion recognition dataset has not been established yet. In this case, how to resolve under-fitting becomes a hot topic.

An emotion recognition model for images such as Chinese paintings is established based on DL approaches to bridge the gap between the universal, low-level visual features and high-level emotional semantics and address the small sample issue of emotion recognition datasets. Afterward, DL overfitting in the case of a small dataset gets improved based on TL (Transfer Learning). In the meantime, a two-layer TL strategy is proposed for emotion recognition of Chinese paintings to resolve under-fitting during cross-domain transfer.

### DL Approaches for Chinese Painting Emotion Recognition

**FIGURE 2:**
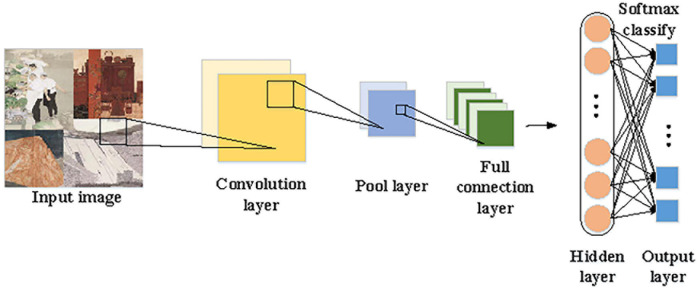
Procedures of CNN processing and recognizing Chinese paintings.

While appreciating Chinese paintings, people often evaluate art pieces regarding artistic conception, charm, and drawing techniques. If emotions in Chinese paintings can be decomposed and analyzed quantitatively, people will understand the way Chinese paintings express emotions and thus better appreciate the beauty of Chinese paintings. Besides, emotion visualization can intuitively demonstrate the digital laws of emotions behind Chinese paintings, greatly enlarging the effect and quality of cultural and artistic learning, analysis, and training (Wang et al., 2017). CNN, RNN (Recurrent Neural Network), and LSTM (Long Short-Term Memory) are standard image recognition algorithms. In the present work, CNN is selected for emotion recognition. CNN is a feedforward neural network, which often contains multiple layers in different types, such as convolutional layers, fully connected layers, and pooling layers (Haberl et al., 2018; Kim et al., 2020). Figure 2 visualizes the procedures of CNN processing and recognizing Chinese paintings.

Normally, CNN performs convolution operations in multiple dimensions. Suppose the input is a 2D matrix *I*; in that case, there is a 2D kernel *K* satisfying the following equation:


(1) $$S\,\left(i,j\right)=\left(I\cdot K\right)\,\left(i,j\right)=\sum_{m}\sum_{n}I\,\left(m,n\right)\,K\,\left(i-m,j-n\right)$$

In (1), (*i, j)* describes the matrix dimension, and *(m, n)* refers to the matrix order. Convolution can be exchanged and equivalently written as the following equation:


(2) $$S\,\left(i,j\right)=\left(I\cdot K\right)\,\left(i,j\right)=\sum_{m}\sum_{n}I\,\left(i-m,j-n\right)\,K\,\left(m,n\right)$$

The flipped convolution kernel, as opposed to the input, gives interchangeability features to the convolution operation; the input index is increasing, while the kernel index is decreasing. The only goal of kernel flipping is to achieve exchangeability; although this feature is helpful in proofs, it is not that significant to neural networks. Instead, many neural network libraries possess a function called CC (Cross-Correlation) (Lin et al., 2018), almost the same as the convolution operation but cannot flip the kernel:


(3) $$S\,\left(i,j\right)=\left(I\cdot K\right)\,\left(i,j\right)=\sum_{m}\sum_{n}I\,\left(i+m,j+n\right)\,K\,\left(m,n\right)$$

Convolutional neural network classifies pixels of the original Chinese paintings before the up-sampling operation that continuously scales the image to the original size. The final output is the up-sampled image, so that each pixel in the output image can be forecasted. In particular, the maximal image pixel is found in all the images finally obtained. AlexNet has more layers and stronger learning capability among various CNNs; thus, it is selected in the present work to reduce the calculation amount and strengthen the generalization performance of the algorithm (Jia et al., 2019). Suppose that $A_{i}^{\left(l\right)}$ denotes the output of the *l*-th convolutional layer in CNN, *A*^(0)^ describes the Chinese painting input; in that case, its *i*(1≤*i*≤*M*^(*l*)^)-th feature map can be calculated through:


(4) $$A_{i}^{\left(l\right)}=\sigma \,\left(\sum_{j=1}^{M^{\left(l-1\right)}}A_{j}^{\left(l-1\right)}⊗W_{i,j}^{\left(l\right)}+b_{i}^{\left(l\right)}\right)$$

In (4), *M*^(*l*)^ refers to the total number of feature maps of the *l*-th convolutional layer, *W* represents the weight of the convolution kernel, *b* is the bias, _⊗_ signifies the convolution operation, and σ(⋅) demonstrates ReLU (Rectified Linear Unit) (Gu et al., 2019). ReLU can fix vanishing and exploding gradient and enhance the network’s expression. In the pooling layer, standard pooling strategies are Max-Pooling and Average-Pooling. AlexNet often employs the Max-Pooling strategy. After the convolutional layer and the pooling layer map the original data to the hidden layer characteristic space, multiple fully connected layers are connected to map the learned feature representation to the sample’s label space. AlexNet has three fully connected layers; their equations are:


(5) $$A_{i}^{\left(6\right)}=\sigma \,\left(\sum_{j=1}^{n^{\left(5\right)}}W_{i,j}^{\left(6\right)}\times F_{j}\,\left(A^{\left(5\right)}\right)+b_{i}^{\left(6\right)}\right)$$

(6) $$A_{i}^{\left(7\right)}=\sigma \,\left(\sum_{j=1}^{n^{\left(6\right)}}W_{i,j}^{\left(7\right)}\times A^{\left(6\right)}+b_{i}^{\left(7\right)}\right)$$

(7) $$A_{i}^{\left(8\right)}=\varphi \,\left(\sum_{j=1}^{n^{\left(7\right)}}W_{i,j}^{\left(8\right)}\times A^{\left(7\right)}+b_{i}^{\left(8\right)}\right)$$

In (5) ∼ (7), *n*^(*l*)^ refers to the number of neurons in the *l*-th layer, *F*(⋅) refers to the tiling operation in which the result of the last convolutional layer is expanded into a 1D vector, φ(⋅) signifies SoftMax that predicts the probability that the input vector belongs to each emotion category, and its equation is:


(8) $$\varphi_{j}\,\left(X\right)=\frac{e^{X_{j}}}{\sum_{k}e^{X_{k}}}$$

**FIGURE 3:**
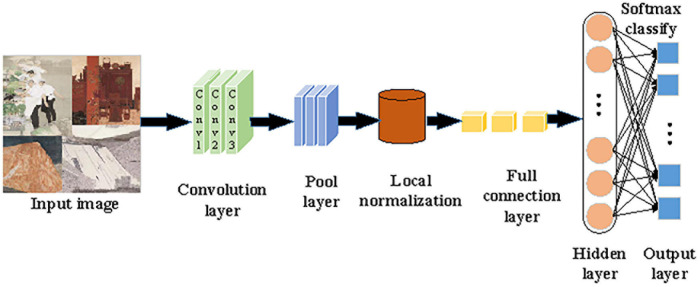
Procedures of the improved AlexNet processing and recognizing Chinese paintings.

In (8), *j* represents the *j*-th element of the input vector *X*, and *k* denotes the total number of sample categories. Furthermore, AlexNet’s convolutional layer is improved. The operation of “local normalization before pooling” is advanced to “pooling before local normalization.” This improvement brings two benefits. First, the generalization ability of AlexNet can be enhanced while the over-fitting can be weakened, which greatly shortens the training time. Second, pooling before local normalization can retain useful information, weaken redundant information, and accelerate the convergence of the proposed algorithm, highlighting the superiority of overlapping Max-Pooling. Figure 3 presents the procedures of the improved AlexNet processing and recognizing Chinese paintings.

Removing redundant information from feature data is a necessary step before an emotion recognition task, especially when the amount of original modality data is particularly huge. Traditionally, text information and image information can be extracted from images for emotion recognition. However, emotion recognition of Chinese paintings is a binary classification task. Only using image information can accurately determine the negativity and positivity of the user’s current emotion. Hence, image information occupies more weight than text information. If text information appears in this case, it will be regarded as subordinate redundant information. A process to remove redundant information in the modality feature, retain the weighted feature in the emotion recognition task, and reduce the calculation cost and resource wastes is extremely meaningful and remarkable. There are three well-known feature selection methods: packaging, embedding, and filtering. The packaging method comprises a learning algorithm that can evaluate the accuracy performance of a subset of candidate features, providing better solutions than the other two methods. SVM-RFE (Support Vector Machine-Recursive Feature Elimination) is a standard packaging method (Spoorthi et al., 2018; Nalepa et al., 2019; Sukumarana and Mb, 2019). Suppose a given training sample set as {*x*_*i*_,*y*_*i*_},*x*_*i*_ ∈ *R^d^*,*y*_*i*_ ∈ {−1,1},*i* = 1,…,*n*; in that case, the decision function of linear SVM is:


(9) f⁢(x)=w*x+b

In (9), *w* represents the weight, and *b* denotes the bias term. The boundary *M* is proved to be only 2/|*w*|. Hence, the maximum boundary is equivalent to minimizing ||*w*||^2^ under constraints. The dual form of the LaGrange problem can be expressed as:


(10) $$L_{D}=\sum_{i=1}^{n}\partial_{i}-\frac{1}{2}\,\sum_{j=1}^{n}\partial_{i}\partial_{j}y_{i}\,y_{j}\,x_{i}\,x_{j}$$

In (10), ∂*_i_* refers to the LaGrange multiplier. The solution to ∂*_i_* can be calculated by maximizing *L*_*D*_ under constraints ∂*_i_*≥0 and $\sum_{i=1}^{n}\partial_{i}y_{i}=0$. Samples corresponding to non-zero _∂_ are called support vectors. The weight vector w can be obtained by the following equation:


(11) $$w=\sum_{i=1}^{n}\partial_{i}y_{i}\,x_{i}$$

Then, the ranking criterion of the pixel feature *k* of Chinese paintings is the square of the *k*-th element of *w*:


(12) $$J\,\left(k\right)=w_{k}^{2}$$

SVM-RFE trains all SVM features to calculate their weights, which is pretty cumbersome. Chi Square Test and information gain are two widespread filtering methods. During feature extraction, Chi Square Test measures the dependency between features and categories. The higher the score, the more dependent the categories are on the given features. Thus, features with lower scores have less information and should be deleted. Suppose that a feature in a Chinese painting is independent of the final classification; in that case, the Chi Square Test can be defined as:


(13) $$C\,H\,I\,\left(t,c_{i}\right)=\frac{N\times {\left(A\,D-B\,C\right)}^{2}}{\left(A+C\right)\times \left(B+D\right)\times \left(A+B\right)\times \left(C+D\right)}$$

(14) $$C\,H\,I_{\max}\,\left(t\right)=\max\left(C\,H\,I\,\left(t,c_{i}\right)\right)$$

In (13) and (14), *A* refers to the number of documents with feature *t* and belonging to category *c*_*i*_, *B* describes the number of documents with feature *t* but not belonging to category *c*_*i*_, *C* refers to the number of occurrences of *c*_*i*_ without *t*, *D* signifies the frequency where neither *c*_*i*_ nor *t* appears, and *N* refers to the total number of instances in the document set, *N = A+B+C+D*. If *t* and *c*_*i*_ are independent, the *Chi* statistic will be zero.

Information gain measures the information obtained after the feature values in the document are known. The higher the information gain, the better the ability to distinguish different categories. The information can be calculated by capturing the uncertainty entropy of the probability distribution of a given category. Suppose there are *m* categories: *C* = {*c*_1_,*c*_2_,⋯,*c*_*m*_}; in that case, the following equation can be deduced based on entropy:


(15) $$H\,\left(C\right)=-\sum_{i=1}^{m}p\,\left(c_{i}\right)\,\log_{2}p\,\left(c_{i}\right)$$

In (15), *p*(*c*_*i*_) denotes the probability of how many documents are in category *c*_*i*_. Suppose that the attribute *A* in Chinese paintings has *n* different values: *A* = {*a*_1_,*a*_2_,⋯,*a*_*m*_}; in that case, the entropy after examining attribute *A* can be defined as follows:


(16) $$H\,\left(C|A\right)=\sum_{j=1}^{m}\left(-P\,\left(a_{j}\right)\,\sum_{i=1}^{m}p\,\left(c_{i}|a_{j}\right)\,\log_{2}p\,\left(c_{i}|a_{j}\right)\right)$$

In (16), *P*(*a*_*j*_) suggests the probability of how many documents contain the attribute value *a*_*j*_, and *p*(*c*_*i*_|*a*_*j*_) describes the probability of the number of documents containing the attribute value *a*_*j*_ in category *c*_*i*_. Based on the above definition, the information gain of an attribute is exactly the difference between the entropy values before and after examining the attribute, which can be expressed as:


(17) I⁢G⁢(A)=H⁢(C)-H⁢(C|A)

Therefore, during sentiment analysis, the filtering method can select a subset of features from the original features, use statistical measures to rank the available features, and automatically filter out those features whose scores are lower than a predetermined threshold. More importantly, it can assess feature subsets without learning algorithms. In short, this method is easy to design and does not require substantial computing resources, showing significant advantages for large datasets.

### The Multimodal Emotion Recognition Algorithm for Chinese Paintings at the Thirteenth National Exhibition of Fines Arts in China Based on Chi Square Test and Improved AlexNet

**FIGURE 4:**
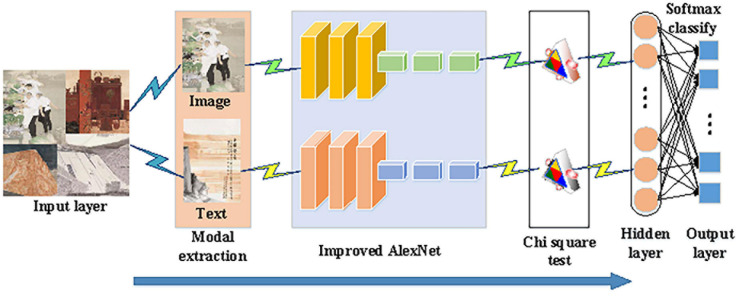
Procedures of the proposed multimodal emotion recognition algorithm based on Chi Square Test and improved AlexNet processing Chinese paintings at the Thirteenth National Exhibition of Fines Arts in China.

A multimodal emotion recognition algorithm for Chinese paintings is designed based on improved AlexNet and Chi Square Test. In particular, because it has more layers and stronger learning capability than other CNNs, AlexNet is chosen in the present work and gets improved to solve the “semantic gaps” in recognizing emotions from images such as Chinese paintings. Furthermore, Chi Square Test removes data redundancy and data noise in each modality of Chinese paintings and captures the internal connections among the modalities. This algorithm not only addresses the above concerns but also saves calculation costs and improves emotion recognition accuracy. Figure 4 below explains its procedures.

The proposes algorithm obeys the following two principles: (1) thoroughly utilizing different modality data, avoiding any wastes, and (2) choosing the most relevant features in high-dimensional spaces to discover the internal connections among features. It can provide more excellent classification accuracy than other algorithms.

During training, one local normalization layer is attached after AlexNet’s pooling layer to standardize the feature map $c_{t}^{l}\,\left(i,j\right)$:


(18) $$c_{t}^{l}\,\left(i,j\right)=a_{t}^{l}\,\left(i,j\right)/{\left(k+\alpha \,\sum_{\max\left(0,t-m/2\right)}^{\min\left(N-1,t+m/2\right)}{\left(a_{t}^{l}\,\left(i,j\right)\right)}^{2}\right)}^{\beta }$$

In (18), *k*,α,β,*m* are hyperparameters valuing 2, 0.78, 10^–4^, and 7, respectively, and *N* is the total number of convolution kernels in the *l*-th convolutional layer. To prevent “gradient dispersion” (Xu and Li, 2021) ReLU is employed to activate the convolution output $S_{t}^{l}\,\left(i,j\right)$:


(19) $$y_{t}^{l}\,\left(i,j\right)=f\,\left(S_{t}^{l}\,\left(i,j\right)\right)=\max\left\{0,S_{t}^{l}\,\left(i,j\right)\right\}$$

In (19), *f()* represents ReLU. To avoid over-fitting in the fully connected layer, the dropout parameter is set to 0.5.

While improving the generalization ability, neurons *C^ll^* in the fully connected layer are discarded and output, $r_{j}^{l}∼b\,e\,r\,n\,o\,u\,l\,l\,i\,\left(d\,p\right),{\tilde{C}}^{l}=r^{l}\,C^{l}$ ; in that case, the *i*-th neuron input in the fully connected layer $Z_{i}^{l+1}$ is $W_{i}^{l+1}\,{\tilde{C}}^{l}+b_{i}^{l+1}$, where the *i*-th neuron input in the next fully connected layer $C_{i}^{l}$ is $f\,\left(Z_{i}^{l}\right)$, namely $\max\left\{0,Z_{i}^{l}\right\}$. Eventually, the input *q^i^* of the *i*-th neuron in the fully connected layer can be obtained:


(20) $$q^{i}=s\,o\,f\,t\,\max\left(Z_{i}^{8}\right)=\frac{e^{Z_{i}^{8}}}{\sum_{j=1}^{12}e^{Z_{i}^{8}}}$$

Here, the cross-entropy loss function suitable for classification is taken as the algorithm’s error function, and the equation is:


(21) $$L\,o\,s\,s=\sum_{i=1}^{K}y_{i}\cdot \log\left(p_{i}\right)$$

(22) $$p_{i}=\frac{\exp\left({\tilde{y}}_{i}\right)}{\sum_{i=1}^{K}\exp\left({\tilde{y}}_{j}\right)}$$

In (21) and (22), *N* signifies the number of categories, *y*_*i*_ represents the actual category distribution of samples, ${\tilde{y}}_{i}$ describes the network output, and *p*_*i*_ denotes the result after the SoftMax classifier. SoftMax’s input is an *N*-dimensional real number vector, denoted as *x*. Its equation is:


(23) $$\xi \,{\left(x\right)}_{i}=\frac{e^{x_{i}}}{\sum_{n=1}^{N}e^{x_{i}}},i=1,2,\ldots ,N$$

Essentially, SoftMax can map an *M*-dimensional arbitrary real number vector to an *M*-dimensional vector whose values all fall in the range of (0,1), thereby normalizing the vector. To reduce the computational amount, the output data volume is reduced to 2^8^ through μ companding conversion, that is, μ = 255, thereby improving the algorithm’s forecasting efficiency.


(24) $$f\,\left(x_{t}\right)=s\,i\,g\,n\,\left(x_{t}\right)\,\frac{\ln\left(1+\mu \,\left|x_{t}\right|\right)}{\ln\left(1+\mu \right)},\left|x_{t}\right|<1$$

The proposed algorithm is trained through learning rate updating using the polynomial decay approach (Poly) (Cai et al., 2020). The equation is:


(25) $$i\,n\,i\,t\,\_\,l\,r\times {\left(1-\frac{e\,p\,o\,c\,h}{\max\_\,e\,p\,o\,c\,h}\right)}^{p\,o\,w\,e\,r}$$

In (26), the initial learning rate *init_lr* is 0.0005 (or 5e^–4^), and power is set to 0.9. The Weighted Cross-Entropy (WCE) is accepted as the cost function to optimize the algorithm training process. Suppose that *z*_*k*_(*x*,θ) describes the unnormalized logarithmic probability of pixel *x* in the *k*-th category under the given network parameter θ. In that case, the SoftMax function *p*_*k*_(*x*,θ) is defined as:


(26) $$p_{k}\,\left(x,\theta \right)=\frac{\exp\left\{z_{k}\,\left(x,\theta \right)\right\}}{\sum_{k^{\prime }}^{K}\exp\left\{z_{k^{\prime }}\,\left(x,\theta \right)\right\}}$$

In (27), *K* represents the total number of image categories. During forecasting, once equation (26) reaches the maximum, pixel *x* will be labeled as the *k*-th category, namely k* = *argmax*{*P*_*k*_(*x*,θ)}. A semantic segmentation task needs to sum the pixel data loss in each input mini-batch (Li et al., 2018; Guo et al., 2019). Suppose that *N* denotes the total number of pixels in the training batch of image data, *y*_*i*_ refers to the real semantic annotation of the pixel *x*_*i*_, and *p*_*k*_(*x*_*i*_,θ) describes the forecasted probability of pixel *x*_*i*_ belonging to the *k*-th semantic category, that is, the log-normalized probability, abbreviated as *p*_*ik*_. In that case, the training process aims to find the optimal network parameter θ* by minimizing the WCE loss function ℓ(*x*,θ), denoted as $\theta *=\min_{\theta }\ell \left(x,\theta \right)$. Training samples with unbalanced categories in brain images usually make the network emphasize some easily distinguishable categories, resulting in poor recognition on some more difficult samples. In this regard, the Online Hard Example Mining (OHEM) strategy (Wen et al., 2019) is adopted to optimize the network training process. The improved loss function is:


$$\ell \,\left(x,\theta \right)=-\frac{1}{\sum_{i=1}^{N}\sum_{k=1}^{K}\delta \left(y_{i}=k,p_{i\,k}<\eta \right)}$$

(27) $$\sum_{i=1}^{N}\sum_{k=1}^{K}\delta \left(y_{i}=k,p_{i\,k}<\eta \right)\log p_{i\,k}$$

In (27), η ∈ (0,1] refers to the predefined threshold, and δ(⋅) describes the symbolic function, which will value 1 if the condition is met and 0 otherwise. The weighted loss function for brain image fusion is defined as:


(28) $$\ell \,\left(x,\theta \right)=-\sum_{i=1}^{N}\sum_{k=1}^{K}w_{i\,k}\,q_{i\,k}\,\log p_{i\,k}$$

In (28), *q*_*ik*_ = *q*(*y*_*i*_ = *k*|*x*_*i*_) denotes the true label distribution of the *k*-th category of the pixel *x*_*i*_, and *w*_*ik*_ refers to the weighting coefficient. The following strategy is employed during training:


(29) $$w_{i\,k}=\frac{1}{\ln\left(c+p_{i\,k}\right)}$$

In (29), *c* is an additional hyperparameter, set to 1.10 based on experience in the simulation experiments of the present work.

**FIGURE 5:**
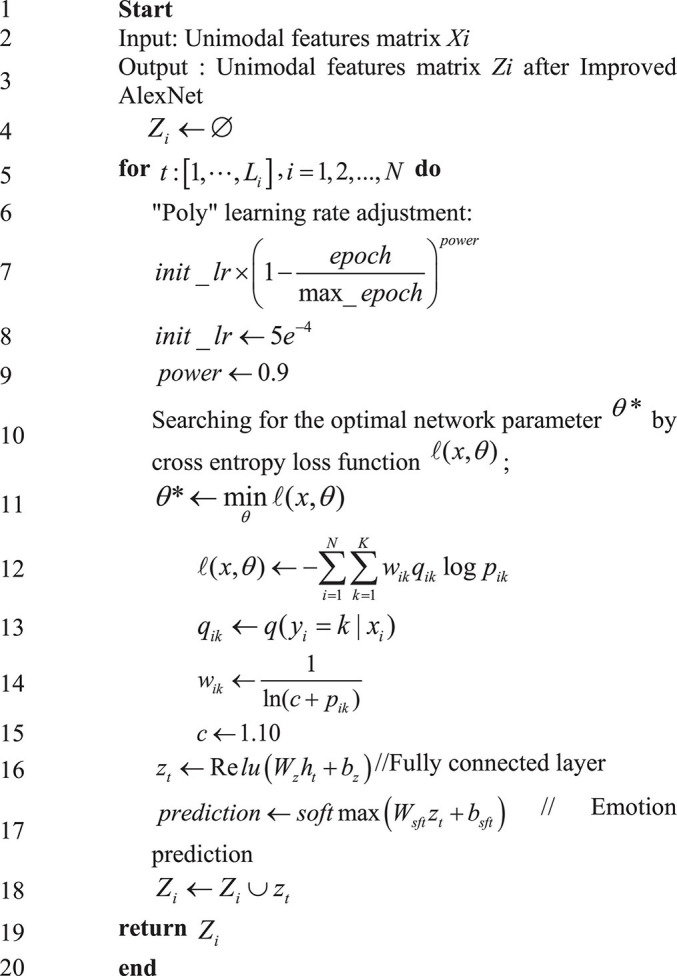
Procedures of the proposed algorithm based on Chi Square Test and improved AlexNet.

Procedures of the proposed algorithm are illustrated in Figure 5.

### Simulation Experiments

MATLAB is adopted in the present work to validate the performance of the proposed algorithm. Image data utilized in the simulation experiments come from Chinese paintings at the Thirteenth National Exhibition of Fines Arts in China; they are divided into a training dataset and a test dataset in 7:3. The ratio of each data type in the two datasets shall be consistent. Hyperparameters of the neural network are set as follows: 0.5 dropout, 300 fully connected layer, 120 iterations, and 40 mini-batch to avoid over-fitting. Some state-of-art algorithms are included for performance comparison, including LSTM (Wen et al., 2019), CNN (Ul Haq et al., 2019), RNN (Zhang et al., 2018), AlexNet (Sajjad and Kwon, 2020), and DNN (Jain et al., 2020). Experimental environment configuration includes software and hardware. Regarding software, the operating system is Linux 64bit, the Python version is 3.6.1, and the development platform is PyCharm. Regarding hardware, the Central Processing Unit (CPU) is Intel Core i7-7700@4.2GHz 8 Cores, the internal memory is Kingston DDR4 2,400 MHz 16G, and the Graphics Processing Unit (GPU) is NVIDIA GeForce 1060 8G.

Performance evaluation metrics include Accuracy, Precision, Recall, and F1 score, calculated through the following equations:


(30) $$A\,c\,c=\frac{\sum_{i=1}^{l}\frac{T\,P_{i}+T\,N_{i}}{T\,P_{i}+F\,P_{i}+T\,N_{i}+F\,N_{i}}}{l}$$

(31) $$\mathrm{Pr}e\,c\,i\,s\,i\,o\,n=\frac{\sum_{i=1}^{l}\frac{T\,P_{i}}{T\,P_{i}+F\,P_{i}}}{l}$$

(32) $$R\,e\,c\,a\,l\,l=\frac{\sum_{i=1}^{l}\frac{T\,P_{i}}{T\,P_{i}+F\,N_{i}}}{l}$$

(33) $$F\,1=\frac{2\,\text{Pr}\,e\,c\,i\,s\,i\,o\,n\cdot R\,e\,c\,a\,l\,l}{\text{Pr}\,e\,c\,i\,s\,i\,o\,n+R\,e\,c\,a\,l\,l}$$

In (30) ∼ (33), *TP* represents the number of positive samples forecasted to be positive, *FP* represents the number of negative samples forecasted to be positive, *FN* represents the number of positive samples forecasted to be negative, and *TN* represents the number of negative samples forecasted to be negative. Accuracy measures the overall classification accuracy, that is, the proportion of samples that forecasted correctly. Recall measures the coverage of positive samples, that is, the proportion of correctly classified positive samples to the total number of positive samples. Precision represents the proportion of examples classified as positive examples to actual positive examples. The most commonly used method is F1 score, which is the weighted harmonic average of precision and recall.

Meanwhile, the emotion recognition efficiency of the algorithm is analyzed from the perspective of SpeedUp. SpeedUp is the ratio of time consumed by the same task running in single processor system and parallel processor system, used to measure the performance and effect of parallel system or program parallelization.

## Results and Discussion

### Analysis of Forecasting Performance

**FIGURE 6:**
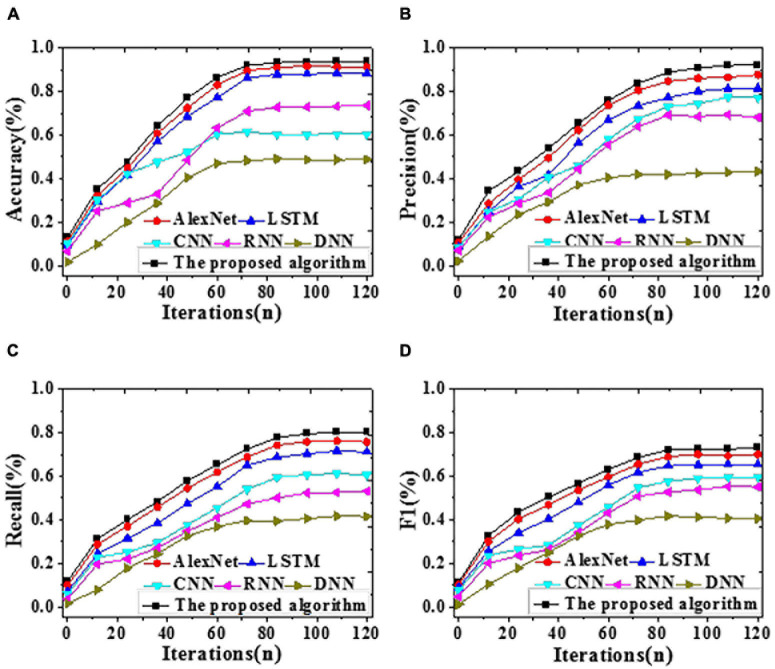
The recognition accuracy of different algorithms with iteration on the training dataset [**(A)** Accuracy; **(B)** Precision; **(C)** Recall; **(D)** F1 score].

**FIGURE 7:**
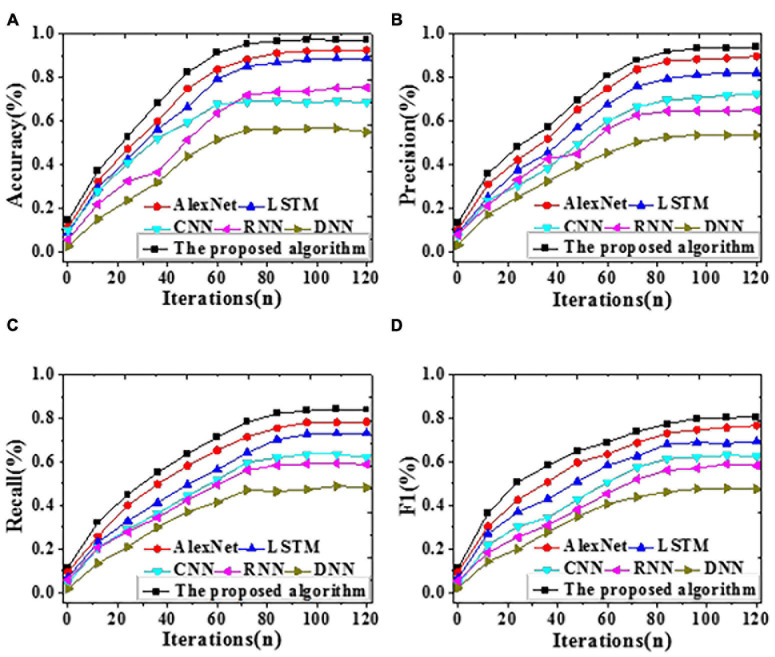
The recognition accuracy of different algorithms with iteration on the test dataset [**(A)** Accuracy; **(B)** Precision; **(C)** Recall; **(D)** F1 score].

To analyze the forecasting performance, the proposed algorithm, LSTM, CNN, RNN, AlexNet, and DNN are included for comparative simulation. Figures 6, 7 visualize the results of forecasting accuracy (Accuracy, Precision, Recall, and F1 score). Tables 1, 2 illustrate the results of time durations required for training and testing.

**TABLE 1:** Time duration required by different algorithms on the training dataset(s).

	**Epochs**		
	**1.00**	**60.00**	**120.00**
The proposed algorithm	90.50	57.91	54.97
AlexNet	90.50	63.44	60.09
LSTM	90.25	67.97	65.87
CNN	91.00	71.49	70.90
RNN	91.51	75.51	75.42
DNN	90.50	80.28	80.20

**TABLE 2:** Time duration required by different algorithms on the test dataset(s).

	**Epochs**		
	**1**	**60**	**120**
The proposed algorithm	57.81	25.04	23.74
AlexNet	58.15	26.13	24.44
LSTM	57.31	31.03	27.90
CNN	57.65	34.89	29.92
RNN	58.49	39.26	36.81
DNN	59.66	44.64	42.69

As shown in Figure 6, on the training dataset, the proposed algorithm can provide an Accuracy of 92.23%, reaching an improvement of at least 4.56% over other algorithms. Remarkably, its Precision, Recall, and F1 score are also the best, at least 3.03% higher than others. To sum up, the proposed algorithm can provide a notably better forecasting performance than DNN, LSTM, AlexNet, RNN, and CNN on the training dataset.

According to Figure 7, on the test dataset, the proposed algorithm can provide an Accuracy of 97.11%, reaching an improvement of at least 4.66% over other algorithms. Remarkably, its Precision, Recall, and F1 score are also the best, at least 0.44% higher than others. Hence, the proposed algorithm can provide a remarkably better forecasting performance than DNN, LSTM, AlexNet, RNN, and CNN on the test dataset. Tables 1, 2 below illustrate the results of time durations required on the training and test datasets.

Time durations required by all algorithms first decrease sharply then tend to stabilize with epochs; that is, the algorithms converge. In particular, the proposed algorithm requires a training duration of 54.97 s and a testing duration of 23.74 s, remarkably shorter than other algorithms. The proposed algorithm takes less time to make forecasts because of its enhanced generalization ability and accelerated algorithm convergence. In the meantime, the Chi Square Test is specific to emotion recognition, which reduces the time required again. To sum up, the proposed algorithm can achieve higher forecasting accuracy more quickly than other simulated algorithms.

### Analysis of Acceleration Efficiency

**FIGURE 8:**
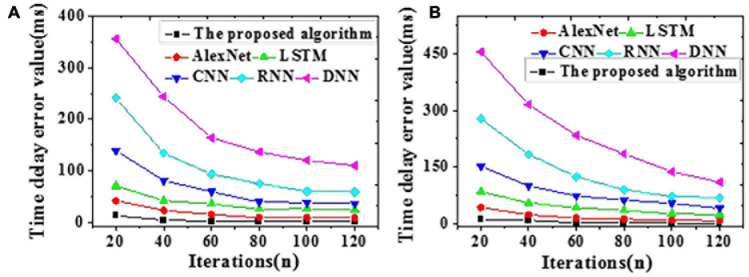
The time delay error of different algorithms for Chinese painting emotion recognition [**(A)** The test dataset; **(B)** The training dataset].

**FIGURE 9:**
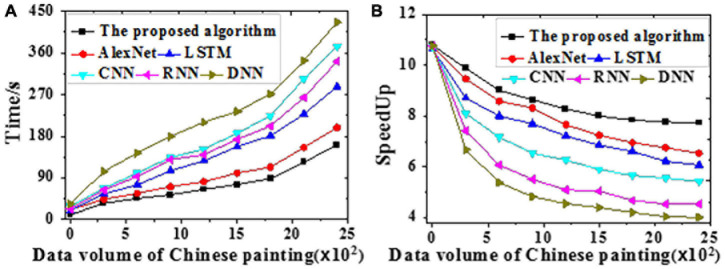
The time required and speedup ratio of different models under different data volumes on the training dataset [**(A)** Time required; **(B)** Speedup ratio].

**FIGURE 10:**
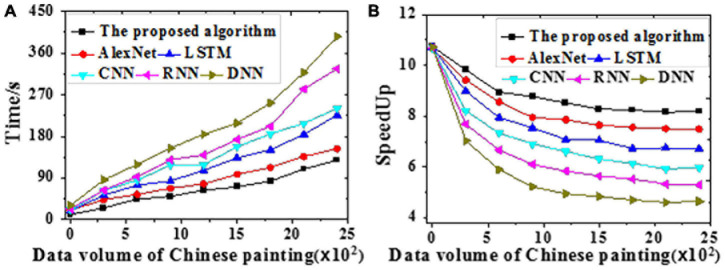
The time required and speedup ratio of different models under different data volumes on the test dataset [**(A)** Time required; **(B)** Speedup ratio].

The acceleration efficiency is tested on the training and the test datasets, respectively. Results of time delay error for Chinese painting emotion recognition are visualized in Figure 8. The time required and speedup ratio of different algorithms under different data volumes are presented in Figures 9, 10.

As shown in Figure 8, errors gradually reduce with iterations on both the training and the test datasets. DNN provides the longest time delay, reaching 455.91 and 356.21 ms, respectively. In contrast, the proposed algorithm provides a time delay approaching zero, the smallest among all simulated algorithms.

According to Figures 9, 10, the proposed algorithm is less sensitive to data growth than other algorithms. Hence, it is suitable to process massive data; the larger the data volume, the higher the speedup ratio, and the greater the acceleration ratio. All algorithms provide slightly better acceleration efficiencies on the test dataset than the training dataset probably because of the absent emotion recognition and analysis path for Chinese paintings during training. Through the adaptive learning, neural networks can process massive amounts of emotion data in Chinese paintings, which remarkably increases the efficiency. To sum up, the proposed algorithm can better recognize and classify human affects in Chinese paintings than other algorithms.

Aiming at the abstract phenomenon of emotion expressed in the field of Chinese painting, the study proposes a multimodal emotion recognition model of Chinese painting based on improved AlexNet neural network and chi square test. The simulation analysis reveals that the recognition and prediction accuracy is significantly higher than that of LSTM, CNN, RNN, AlexNet and DNN model algorithms proposed by scholars in related fields. Among them, the accuracy of the proposed algorithm in the test set reaches 97.11%, and the required time is only 23.74 s. This may be because the improved AlexNet neural network proposed not only enhances the generalization ability, but also accelerates the convergence rate of the model training process. Moreover, the chi square test is targeted for emotion recognition, which reduces the time required for emotion recognition again. Regarding the recognition efficiency, it is also obvious that the acceleration ratio of the proposed algorithm is higher. This may be because the model has not formed an emotion recognition and analysis path corresponding to Chinese painting in the training process. After autonomous learning in the training process, the neural network can better analyze many emotions of Chinese painting, and the efficiency has been significantly improved. Therefore, the algorithm proposed has a good effect on emotion recognition of Chinese painting.

## Conclusion

Art such as Chinese painting aims to express the esthetics and emotion of works through visual art elements such as color, line, and shape. Thus, emotion recognition of images is not only conducive to the management of art information, but also can promote the popularization and promotion of art. To solve “semantic gap” in the emotion recognition task of images such as traditional Chinese painting, this study constructs a multimodal emotion recognition model of Chinese painting based on improved AlexNet neural network and chi square test. The simulation reflects that the emotion recognition accuracy of the improved AlexNet neural network combined with chi square test algorithm model proposed reaches 92.23 and 97.11% in the training set and test set, respectively. The training and test time are stable at about 54.97 and 23.74 s, and the acceleration efficiency is obviously better than other algorithms, which can provide experimental reference for the management, popularization, and promotion of art information in the later stage. However, there are still some deficiencies. First, due to the great differences in the characteristics of traditional Chinese painting in different techniques and content categories, it is difficult to use the same algorithm to identify emotion. In the future, the emotion recognition algorithm for various categories of traditional Chinese painting will be further discussed. Additionally, the mathematical model of traditional Chinese painting emotion will be established, and the traditional Chinese painting emotion will be calculated and processed more accurately to create greater value in the digital management and protection of national quintessence. Second, this study models and analyzes the emotion of images such as Chinese painting in the 13th National Exhibition of Fine Arts. But it is not clear to what extent the proposed method and its theory can be applied to other types of painting and natural images. Therefore, future work will focus on evaluating the effectiveness of this method except for the field of various categories of art.

## Data Availability Statement

The original contributions presented in the study are included in the article/supplementary material, further inquiries can be directed to the corresponding author/s.

## Author Contributions

JL and DC designed the multi-modal emotion recognition algorithm of Chinese paintings. ZZ and NY conducted the evaluation and analysis of experimental data, compared other algorithms include LSTM, CNN, RNN, AlexNet, and DNN under the guidance of ZL. All authors participated in the process of writing and revising the manuscript.

## Conflict of Interest

The authors declare that the research was conducted in the absence of any commercial or financial relationships that could be construed as a potential conflict of interest.

## Publisher’s Note

All claims expressed in this article are solely those of the authors and do not necessarily represent those of their affiliated organizations, or those of the publisher, the editors and the reviewers. Any product that may be evaluated in this article, or claim that may be made by its manufacturer, is not guaranteed or endorsed by the publisher.
